# How does high-commitment work systems stimulate employees’ creative behavior? A multilevel moderated mediation model

**DOI:** 10.3389/fpsyg.2022.904174

**Published:** 2022-08-08

**Authors:** Min Zhang, Zhihong Chen, Lijing Zhao, Xiang Li, Zhi Zhang, Xufan Zhang

**Affiliations:** ^1^Economics and Management School, Nantong University, Nantong, China; ^2^Institute for International Students, Nanjing University, Nanjing, China; ^3^School of Management, Hainan University, Haikou, China; ^4^Ginling College, Nanjing Normal University, Nanjing, China

**Keywords:** high-commitment work systems, employees’ wellbeing, employees’ creative behavior, CEO inclusive leadership, moderated mediation effect

## Abstract

How to effectively stimulate employees’ creative behavior is a hot topic in the field of organizational behavior. Based on conservation of resources theory and substitutes for leadership theory, this paper discusses the impact of high-commitment work systems on employees’ creative behavior and the roles of employees’ wellbeing and CEO inclusive leadership. By constructing a cross-level structural equation model and analyzing the paired data of 86 CEOs, 86 HR managers and 489 employees, the results show that: (1) high-commitment work systems have positive impact on employees’ creative behavior; (2) employee’s wellbeing mediates the process of high-commitment work systems driving employees’ creative behavior; and (3) CEO inclusive leadership negatively moderates the relationship between high-commitment work systems and employees’ wellbeing, and further negatively moderates the indirect effect of high-commitment work systems on employees’ creative behavior through employees’ wellbeing, that is, the lower the level of CEO inclusive leadership is, the stronger the impact of high-commitment work systems on employees’ creative behavior through employees’ wellbeing will be.

## Introduction

The emergence of emerging technologies such as big data and artificial intelligence has hastened the arrival of the digital era. In this context, facing the iterative upgrading of business model, how to comply with the development trend of the times and truly use digital technology to realize industrial upgrading or transformation puts forward higher requirements for enterprises. It is worth noting that active participation of creative talents is necessary so as to continuously update and create information technology and transform digital technology into production factors. In other words, the occurrence of employees’ creative behavior is an important condition for enterprises to realize digital transformation and obtain competitive advantage (Amabile, 1988). For organizations, it is very important to effectively stimulate employees’ potential and creativity. Therefore, how to improve employees’ creative behavior has always been one of the research hotspots of scholars and managers.

Existing literatures show that scholars have carried out a lot of research from the aspects of individual characteristics (such as extroversion) and leadership (such as moral leadership) (Chiang et al., 2015). However, in the organizational context, in addition to leadership, strategic human resource management may also exert a systematic environmental impact on individual creativity and organizational creativity (Tsui et al., 1997). Strategic human resource management is a planned human resource management mode for enterprises to achieve organizational goals. It advocates creating a good system and atmosphere conducive to communication between employees and organizations, so as to furtherly exert a systematic environmental impact on employees’ creativity and organizational creativity (Tsui et al., 1997). Some scholars have found that the implementation of high-performance work systems can increase the information exchange between employees, thus improving employees’ creative performance (Chiang et al., 2015). It is found that scholars pay more attention to how the high-performance work systems affect employees’ creative behavior aimed at improving organizational performance (Pahos and Galanaki, 2022) and the impact mechanism between the high-participation work systems and employees’ creative behavior. Different from the high-performance work systems focusing on production and operation or the high-involvement work systems focusing on work organization, high-commitment work systems pay more attention to the establishment of employees’ relationship, and its impact on employees’ creative behavior is not the same as high-performance work systems or high-involvement work systems (Liao et al., 2021; Aboramadan, 2022). Therefore, this study infers that the high-commitment work systems devoted to establishing employees’ organizational commitment will also have an impact on employees’ creative behavior, because it emphasizes establishing a mutually promoting and high investment employment relationship with employees through the combination of human resource management practices, so as to improve employees’ work attitude and behavior, maximizing the interests of employees and organizations (Collins and Smith, 2006). Unfortunately, the mechanism between these two aspects remains unclear. Based on the above, this study focuses on the possible impact of high-commitment work systems in strategic human resource management on employees’ creative behavior, as well as the mechanism and boundary conditions in between.

Previous researches on the relationship between high-commitment work systems and creative behavior are mostly based on the perspectives of social exchange theory and creativity component theory, etc., (Li et al., 2017). In order to further identify how high-commitment work systems affect employees’ creative behavior, this paper studies the internal mechanism of such impact based on conservation of resources theory. Conservation of resources theory claims that individuals always actively obtain, retain and maintain their own resources. When they have enough resources, it is easy for individuals to achieve their goals, form positive psychological resources, and further help individuals obtain new resources (Hobfoll, 2011), forming a spiral gain of resources. When the organization provides employees with material resources and conditional resources via high-commitment work systems, employees are more willing to work because of their sufficient work resources to achieve their self-worth and life goals, so as to experience positive emotions and feelings, that is, obtain a higher sense of wellbeing (Li and Lin, 2020). As a personal resource, positive emotional experience will further promote employees to make more efforts spontaneously and put forward creative ideas, views and measures more frequently (Chen et al., 2021). According to the above inference, the impact of high-commitment work systems on employees’ creative behavior is likely to be realized through the acquisition of employees’ wellbeing. Therefore, this study selects employees’ wellbeing as the mediating variable of high-commitment work systems affecting employees’ creative behavior, which is believed to play an important mediating role in the relationship between high-commitment work systems and employees’ creative behavior.

Furthermore, this study also attempts to further explore the boundary conditions where high-commitment work systems affect employees’ creative behavior through employees’ wellbeing. In the process of exerting its effectiveness, high-commitment work systems may be affected by other organizational environmental factors coexisting with high-commitment work systems in the organization, such as leadership, which may affect the degree of change to individual behavior and attitude brought by high-commitment work systems (Li et al., 2017). In fact, the impact of top management on the organization and employees cannot be ignored. Leaders can shape the work environment of employees and are a key predictor of employees’ creativity and creative (Hughes et al., 2018). According to substitutes for leadership theory, leaders with similar functions may offset the impact of human resource management practices on individuals (Chuang et al., 2016). Different from the traditional leadership styles such as transformational leadership, authentic leadership and service-oriented Leadership (Wang et al., 2021), inclusive leadership accepts and respects the differences of employees, recognizes and encourages employees’ participation and efforts, and paves the way for subordinates to generate, promote and apply creativity (Choi et al., 2015). Inclusive leadership plays an important role in stimulating employees’ creative behavior (Carmeli et al., 2010; Javed et al., 2019), It should be noted that CEOs with inclusive leadership style help employees expand their personal resources and improve their wellbeing by providing employees with the support of material resources such as knowledge, skills and information. In this process, it may affect employees’ perception of the effectiveness and value of the high-commitment work systems. Hence, this study suggests that CEO inclusive leadership may negatively moderate the relationship between high-commitment work systems and employees’ creative behavior through employees’ wellbeing.

Given all of the above, based on the perspective of conservation of resources theory and substitutes for leadership theory, this study will explore the impact of high-commitment work systems on employees’ creative behavior and the mediation role of employees’ wellbeing in between, and look into CEO’s inclusive leadership as the boundary condition of the path where high-commitment work systems indirectly affect employees’ creative behavior through employees’ wellbeing, with expectation to enrich the research on employees’ creative behavior and provide new ideas and practical guidance for organizations to encourage employees’ creative.

## Theoretical basis and hypothesis

### High-commitment work systems and employees’ creative behavior

High-commitment work systems are a series of human resource management practices applied by enterprises aimed at improving employees’ long-term emotional commitment to the organization, which include strict personnel selection, extensive training, development-oriented performance evaluation, work rotation, employees’ participation in management, team performance evaluation, etc., (Xiao and Björkman, 2006).

Employees’ creative behavior refers to the activity that employees use their knowledge, skills and information to put forward useful and novel ideas and create value-added products and technologies (Zhou, 1998). In organizations, the implementation of high-commitment work systems often provides necessary conditions and resources for employees to carry out creative activities, for the purpose of making employees become the source of improving organizational competitiveness. Specifically, firstly the organization selects employees with high creativity and innovation through strict selection process, laying an important foundation for them to generate diverse ideas and give full play to creativity in their follow-up work (Chen et al., 2018). Secondly, organizations carry out extensive training activities to equip employees with necessary knowledge, skills and information, which make it easier for employees to give birth to creative ideas by connecting with their own knowledge system (Prieto and Pérez-Santana, 2014). At the same time, the adoption of a creativity-linked reward system can stimulate employees’ motivation to generate new ideas and try new behaviors, thereby enhancing employees’ creativity (Jiang J. et al., 2012). In addition, job rotation and job enrichment help employees acquire and integrate knowledge and skills in different jobs (Lin and Sanders, 2017), so as to give better play to their subjective initiative and creativity (Chang et al., 2014). Empirical research shows that the exertion of creativity is based on the existing knowledge and skills in this field, that is, knowledge, skills and information are the necessary conditions for the exertion of creativity (Amabile, 1988). Finally, evaluation based on team performance is conducive to encourage the sharing of knowledge, skills and information among team members, enhance the cooperation between teams (Baer et al., 2010), and provide an open and inclusive environment for the generation of employees’ creative ideas. Similarly, the management mode of emphasizing work autonomy and employees’ participation directly expresses the organization’s trust in employees, and can also make employees feel the organization’s support and encouragement for freedom of thought and creative ideas, thereby effectively arousing employees to make more creative behaviors (Cai et al., 2013). Based on the above, the following hypothesis is suggested:

Hypothesis 1: High-commitment work systems have a significant positive impact on employees’ creative behavior.

### Mediating role of employees’ wellbeing

The conservation of resources theory highlights that individuals will try their best to obtain, retain and maintain valuable resources (Hobfoll, 2011). In the workplace, whether employees can have resources for development and growth depends on whether the organization provides an appropriate environment in terms of systems, policies and opportunities (Hobfoll, 2011), which means that the implementation of high-commitment work systems, including extensive training, information sharing, developmental performance evaluation, decision-making autonomy, teamwork, etc., is necessary social resources at work offered to employees by organization. When these resources are transformed to personal ones (Bakker et al., 2014), the employees’ demands for ability improving, well interpersonal relationship building and personal value realizing can be satisfied (Ehrnrooth and Björkman, 2012), and furtherly their experience of wellbeing at work can be advanced (Li and Lin, 2020). Conversely, when the organization fails to effectively implement the high-commitment work systems, that is, it cannot provide sufficient supporting resources for employees, it means that it increases the probability of employees falling into the resource spiral loss and reduces the possibility of employees obtaining the resource spiral gain. Then it is more likely to have negative experiences such as emotional exhaustion and job burnout at work, and consequently it is not detrimental for the generation of employees’ wellbeing (Hobfoll et al., 2018). Therefrom, this study predicts that high-commitment work systems positively affect employees’ wellbeing.

Employees with a higher sense of wellbeing tend to be more outgoing, active and enthusiastic. They are more likely to win the favor of others at work, establish good interpersonal relationships with colleagues and leaders, and are better at team work. In an organization with team performance as the evaluation standard, employees with a higher sense of wellbeing obtain other people’s support, information sharing and other material and conditional resources, increasing their own resources of knowledge and skills related to creative work (Liu et al., 2018). In addition, it is easier for such employees to establish trust and commitment with the organization and effectively supplement the stock of personal character resources. Meanwhile, the occurrence of creative behavior usually indicates that employees need to consume time, energy and other resources to challenge the current situation and break the routine, accompanied by risk, uncertainty and potential failure (Kleingeld et al., 2011). Compared with low wellbeing employees with less resource stock, high resource stock makes high wellbeing employees have less negative feelings such as emotional exhaustion, stress and anxiety caused by resource consumption in the process of innovation (Hobfoll, 2011), be more confident in controlling the overall situation, overcoming the difficulties and obstacles of breaking through the *status quo* and generating creative behavior, and more likely to engage in creative actions at work and be more willing to devote themselves to the innovation and development of the organization (Chang et al., 2014). In terms of conservation of resources theory, the high-commitment work systems endow employees with a strong sense of wellbeing through the provision of supporting resources, effectively complements the resource stock owned by employees, and promotes employees to have more resource reserves to carry out creative activities, resulting in resource spiral gain. Based on the above, the following hypothesis is suggested:

Hypothesis 2: Employees’ wellbeing plays a mediating role in the relationship between high-commitment work systems and employees’ creative behavior.

### Moderating role of CEO inclusive leadership

Inclusive leadership refers to leaders who show openness, availability and accessibility in the two-way interaction with subordinates, respect and understand employees, give feedback to employees and assume responsibility (Hantula, 2009; Carmeli et al., 2010). It is shown that inclusive leaders are willing to listen to subordinates’ opinions and suggestions, accept new views and ideas, respect and care for subordinates, which is conducive to establishing a good trust relationship with subordinates and providing support for them at work.

As a social resource, CEO inclusive leadership affects the relationship between employees and leaders by providing leadership support, and further affects the predictive effect of high-commitment work systems on employees’ wellbeing. Specifically, the conservation of resources theory proposes that a lost resource can be replaced by another equivalent resource (Hobfoll, 2011). Employees under highly inclusive leadership can acquire more work resources and the trust and emotional support of their superiors, reducing their dependence on the human resources system and obtain higher happiness. In contrast, employees under little inclusive leadership believe that they can get more resources and support from HCWs, leading to a stronger relationship between high commitment work system and employee wellbeing. Specifically, CEOs with openness, availability and accessibility can provide supportive behaviors, such as answering questions and providing employees with necessary knowledge, skills and information, so as to help employees improve their work enthusiasm and achieve their work goals, and gain a positive emotional experience (Sofarelli and Brown, 1998). On the contrary, compared with the supportive behavior provided by high inclusive leadership, employees under low inclusive leadership are more likely to obtain more resources and positive emotional experience from the high commitment work system, employees’ trust and dependence on the high commitment work system are deepened, resulting in a stronger link between the high commitment work system and employees’ wellbeing. In addition, leadership substitution theory shows that organizational factors such as human resource management practice reduce the ability of leaders to affect employees and replace the impact of leaders’ behavior on employees (Stein and Min, 2019). For example, Chuang et al. (2016) studied 162 R&D teams and found that the relationship between human resource management system, team knowledge acquisition and team knowledge sharing is more positive in the case of low authorized leadership. Jiang et al. (2015) also pointed out that service-oriented high-performance work system and service-oriented leadership reduce each other’s positive impact on collective customer knowledge and service atmosphere. Therefore, it is inferred that when the inclusive leadership level of CEO is high, the positive impact of high commitment work system on employee wellbeing will be reduced; and that when the CEO’s inclusive leadership level is low, employees are more likely to appreciate the resources and value provided by the high commitment work system, and employees will have more happy experience at work. In particular, since the human resource management system and leadership behavior can act on individuals together, when the human resource management system and leadership behavior play similar functions, there is an interactive effect (negative moderation), that is, the human resource management system can offset or replace the impact of leaders on employees and organizations (Li et al., 2017). Vice versa, leadership behavior can also be used as a substitute for human resource management system and affect the role of human resource management system in the organization (Chuang et al., 2016). This is because, compared with the informal interaction between the high-commitment work systems and employees, the CEO can have the most direct interaction and contact with employees through leadership behavior. For example, employees can perceive in a more clear way the attention, respect and recognition from the CEO with inclusive leadership style, thereby establishing and cultivating trust between both sides, helping employees effectively reduce their perception of negative events such as risks and pressures at the workplace, and promoting employees to obtain more happy experiences (Ramamoorthy et al., 2005). Obviously, at this moment, the potential value brought by the high-commitment work systems to employees will be reduced (Ramamoorthy et al., 2005), that is, the inclusive leadership of the CEO weakens or offsets the impact of the high-commitment work systems on employees’ attitudes and behaviors to a certain extent. These studies show that when both high commitment work system and inclusive leadership play similar functions on employees, the impact of either party on employees’ wellbeing may be weakened by the other. Moreover, when one of them is missing, it may leave more space for the other to affect employees’ happiness. Based on the above, the following hypothesis is suggested:

Hypothesis 3: CEO inclusive leadership moderates the relationship between high-commitment work systems and employees’ wellbeing. Specifically, the positive promoting effect of high-commitment work systems on employees’ wellbeing will get weakened with the rising of CEO inclusive leadership level.

According to the above research assumption, this study further proposes that the mediating role of employees’ wellbeing is moderated by CEO inclusive leadership. In specific, when the CEO’s inclusive leadership level is low, employees are more likely to perceive the resources and organizational support provided by the organization through the high-commitment work systems, which will strengthen the wellbeing brought by the high-commitment work systems and induce employees to produce more creative behaviors. However, when the CEO’s inclusive leadership level is high, employees are more likely to feel the support and help brought by the leadership, which on the contrary will weaken their perception of resources and support offered at the organizational level. Their wellbeing will be impaired thereby. Meanwhile, it is difficult to extend this impact to the proposal of creative solutions. Based on the above, the following hypothesis is suggested:

Hypothesis 4: CEO inclusive leadership moderates the indirect effect of high-commitment work systems on employees’ creative behavior through employees’ wellbeing. Specifically, with the rising of CEO inclusive leadership level, the mediating effect of high-commitment work systems on employees’ creative behavior through employees’ wellbeing become weaker.

**FIGURE 1:**
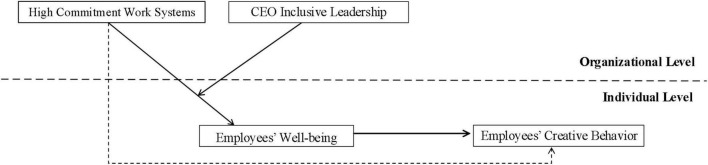
Theoretical model.

To sum up, the theoretical model of this study is shown in Figure 1.

## Research method

### Samples and procedures

In this study, a questionnaire was used to investigate 100 enterprises in Jiangsu, Anhui, Guangzhou, Beijing and other places, including finance, service, manufacturing and other industries. Before collecting the questionnaire, the human resources manager of each enterprise was contacted firstly, helping them understand the purpose and procedure of this survey through communication. Then the questionnaires to the human resources managers of each enterprise were mailed. The employees and CEO were organized to fill in the questionnaires by the human resources managers, and finally the filled questionnaires were returned by mail.

In order to avoid the influence of common method bias, this study collected questionnaires from multiple sources. Among them, the manager of human resources department fills in the items of high-commitment work systems, the CEO fills in the items of inclusive leadership and controlled variables items at enterprise level (enterprise type, enterprise scale and organization age), and the employees fill in those of wellbeing and creative behavior and demographic variables (gender, age, education and tenure). A total of 100 HR manager questionnaires, 100 CEO questionnaires and 600 employee questionnaires were distributed in this study. After collecting and eliminating invalid or obvious duplicate ones, the valid HR manager questionnaires were up to 86, CEO questionnaires were 86, and the valid recovery rate was 86%; while, there were 489 employee questionnaires with a valid recovery rate of 81.5%. The sample characteristics of the survey data are shown in Table 1.

**TABLE 1:** Sample characteristics.

Employee sample characteristics				Enterprise sample characteristics			
Gender (%)		Education (%)		Type (%)		Scale (%)	
Male	54.6%	≤ High School	38.9%	SOE	51.2%	<50	5.8%
Female	45.4%	College	16.4%	Foreign	19.8%	50∼100	9.3%
Age (%)		University	41.9%	Private	15.1%	101∼500	41.9%
≤ 25	18.8%	≥ master	2.9%	Others	14%	501∼1,000	14%
26∼30	33.1%	Tenure by year (%)		Organization age (%)		1,001∼2,000	8.1%
31∼35	25.8%	<1 year	18.4%	<1 year	18.6%	≥ 2,001	20.9%
36∼40	11.2%	1∼3 years	38%	1∼2 years	52.3%		
≥ 41	11%	3∼5 years	20.9%	2∼4 years	23.3%		
		≥ 5 years	22.7%	≥ 4 years	5.8%		

### Variable measurement

The scales used in this study are foreign mature scales, and the four scales are revised in the form of translation and back translation to finalize the questionnaire. All questionnaires were scored with Likert’s seven points, “1–7” represents “completely disagree” to “completely agree.”

### High-commitment work systems

This study adopts the scale established by Xiao and Tsui (2007) which includes 10 items (Xiao and Tsui, 2007). The typical item is “Appraisal of team performance rather than individual performance.” The Cronbachα is 0.79.

### Employees’ wellbeing

This study adopts the scale established by Diener et al. (1985) which includes five items (Diener et al., 1985). The typical item is “I am satisfied with my life.” The Cronbachα is 0.71.

### Employees’ creative behavior

This study adopts the scale established by Scott and Bruce (1994) which includes six items (Scott and Bruce, 1994). The typical item is “Searches out new technologies, processes, techniques, and/or product idea.” The Cronbachα is 0.73.

### CEO inclusive leadership

This study adopts the scale established by Carmeli et al. (2010) which includes nine items (Carmeli et al., 2010). The typical item is “The manager is open to hearing new ideas (openness).” The Cronbachα is 0.83.

### Controlled variables

Referring to previous studies (Chang et al., 2014; Ding et al., 2019), this study mainly selects employees’ gender, age, education, tenure, enterprise type, enterprise scale and organization age as the controlled variables.

## Research results

### Multilevel confirmatory factor analysis

In order to ensure that the variables used in this study have good construct validity, this study conducted multi-level confirmatory factor analysis on the main variables (high-commitment work systems, employees’ wellbeing, employees’ creative behavior and CEO inclusive leadership). By comparing the benchmark model (four-factor model) with several alternative models, it is found that the four factor model has good fitting degree (*χ^2^*/df = 1.279, RMSEA = 0.024, CFI = 0.959, TLI = 0.952, SRMR = 0.030) which is significantly better than other alternative three-factor, two-factor and single-factor models, proving that the four variables involved in this study have good discriminant validity. The results of multilevel confirmatory factor analysis are shown in Table 2.

**TABLE 2:** Results of multilevel confirmatory factor analysis.

Model	*χ^2^*/df	RMSEA	CFI	TLI	SRMR
Four-factor: HCWS, IL, WB, CB	1.279	0.024	0.959	0.952	0.030
Three-factor: HCWS, IL, WB + CB	2.279	0.051	0.810	0.779	0.078
Two-factor: HCWS + IL, WB + CB	3.213	0.067	0.669	0.618	0.078
Single factor: HCWS + IL + WB + CB	8.474	0.124	0.268	0.214	0.129

HCWS, high-commitment work systems; WB, employees’ wellbeing; IL, CEO inclusive leadership; CB, employees’ creative behavior.

### Descriptive statistics

Table 3 describes the mean, standard deviation and correlation coefficient of each variable. Employees’ wellbeing and employees’ creative behavior (γ = 0.349, *P* < 0.01) are significantly positive correlated, which is consistent with our theoretical expectation and provides preliminary support for our hypothesis.

**TABLE 3:** Means, standard deviation and variables correlation.

Individual level	*M*	*SD*	1	2	3	4	5
1. Gender	1.454	0.498					
2. Age	3.697	1.430	−0.120**				
3. Education	2.998	1.093	0.069	−0.160**			
4. Tenure by month	49.875	53.495	−0.031	0.543**	−0.090*		
5. WB	4.323	0.745	0.041	0.032	0.008	0.001	
6. CB	4.356	0.715	−0.030	−0.022	−0.019	−0.021	0.349**

**Organizational level**	***M***	***SD***	**7**	**8**	**9**	**10**	

7. Type	1.919	1.108					
8. Scale	3.720	1.476	−0.223*				
9. Organization age	23.837	17.848	−0.151	0.390**			
10. HCWS	4.578	0.871	−0.041	0.127	0.016		
11. CEO IL	5.023	0.854	−0.158	0.037	−0.034	0.145	

n_employee_ = 489, *N* = 86, **P < 0.01, *P < 0.05.

### Hypothesis test

**FIGURE 2:**
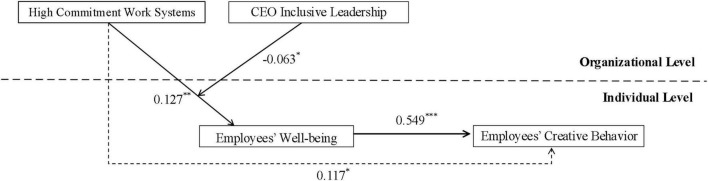
Multilevel SEM model path analysis. ^***^p < 0.001 ^**^p < 0.01, *p < 0.05.

Figure 2 shows the path coefficients between the variables in this study. As shown in Figure 2, high-commitment work systems have a significant positive impact on employees’ creative behavior (γ = 0.117, *P* < 0.05), therefore, hypothesis 1 is supported by the data. Similarly, high-commitment work systems have a significant positive impact on employees’ wellbeing (γ = 0.127, *P* < 0.01), employees’ wellbeing has a significant positive impact on employees’ creative behavior (γ = 0.549, *P* < 0.001), indicating that employees’ wellbeing plays a mediating role between high-commitment work systems and employees’ creative behavior, and hypothesis 2 is supported. Then, this study calculates 20,000 bootstraps to estimate the confidence interval of the indirect effect of high-commitment work systems on employees’ creative behavior through employees’ wellbeing. The results show that the indirect effect of high-commitment work systems on employees’ creative behavior through employees’ wellbeing is 0.070. The test results of Monte Carlo simulation method show that the 95% unbiased signal interval of mediation does not contain zero (95%*LLCI* = 0.021, 95%*ULCI* = 0.135), so hypothesis 2 is further verified.

**FIGURE 3:**
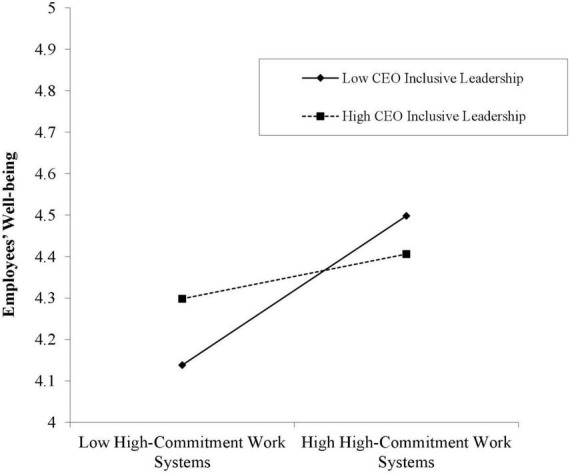
Moderating effect of CEO inclusive leadership on the relationship between high-commitment work systems and employees’ wellbeing.

Hypothesis 3 holds that CEO inclusive leadership will moderate the relationship between high-commitment work systems and employees’ wellbeing. As shown in Figure 2, CEO inclusive leadership negatively moderates the relationship between high-commitment work systems and employees’ wellbeing (γ = −0.063, *P* < 0.05), therefore, hypothesis 3 was supported. In order to reflect the moderating effect of CEO inclusive leadership in a more intuitive way, this study mapped the moderating effect of CEO inclusive leadership on high-commitment work systems and employees’ wellbeing at levels higher and lower than a standard deviation. As shown in Figure 3, compared with CEO inclusive leadership at a high level (γ = 0.065, 95%*LLCI* = −0.011, 95%*ULCI* = 0.142). CEO inclusive leadership at a low level makes the positive effect of high-commitment work systems on employees’ wellbeing stronger (γ = 0.168, 95%*LLCI* = 0.087, 95%*ULCI* = 0.248), and hypothesis 3 was further verified.

This study also uses Monte Carlo simulation to test the moderating mediating effect. In Table 4, the Monte Carlo simulation results show that CEO inclusive leadership can moderate the mediating effect of employees’ wellbeing between high-commitment work systems and employees’ creative behavior, that is, when the level of CEO inclusive leadership is low, the indirect effect of high-commitment work systems on employees’ creative behavior through employees’ wellbeing is 0.098 (95%*LLCI* = 0.033, *ULCI* = 0.181), and the 95% confidence interval does not include 0, which means the effect is significant; while when the CEO’s inclusive leadership level is high, the indirect effect value is 0.036 (95%*LLCI* = −0.014, *ULCI* = 0.096), and the 95% confidence interval contains 0, which does not reach the significant level. The effect value of the difference between groups was −0.062 (95%*LLCI* = −0.014, *ULCI* = 0.096), which indicates that the indirect effect of high-commitment work systems on employees’ creative behavior through employees’ wellbeing is moderated by CEO inclusive leadership, and there is a moderated mediation effect. Therefore, Hypothesis 4 is supported.

**TABLE 4:** Monte Carlo simulation tests the moderated mediating effect.

Dependent variable	CEO IL	Effect value	Standard error	Lower limit	Upper limit
Employees’ CB	High	0.036	0.027	−0.014	0.096
	Low	0.098**	0.037	0.033	0.181
	Difference	−0.062*	0.033	−0.136	−0.005

20,000 bootstrap; **P < 0.01, *P < 0.05.

## Conclusion and discussion

### Result discussion

The occurrence of employees’ creative behavior is often accompanied by many uncertainties and risks, which requires individuals to consume and lose their own resources. Therefore, understanding the occurrence mechanism of employees’ creative behavior is beneficial to the organization to effectively encourage employees to carry out creative activities and improve the organization’s competitiveness. Based on the perspective of conservation of resources theory and substitutes for leadership theory, this study constructs a cross-level model to explore the internal mechanism of high-commitment work systems on employees’ creative behavior. The results show that high-commitment work systems have a positive impact on employees’ creative behavior; employees’ wellbeing plays an mediating role in the relationship between high-commitment work systems and employees’ creative behavior, that is, high-commitment work systems promote employees’ creative behavior by improving employees’ wellbeing; CEO inclusive leadership can significantly negatively moderate the relationship between high-commitment work systems and employees’ wellbeing, and further negatively moderate the process of high-commitment work systems affecting employees’ creative behavior through employees’ wellbeing, which means the lower the level of CEO inclusive leadership is, the stronger the effect of high-commitment work systems affecting employees’ creative behavior through employees’ wellbeing.

### Theoretical contribution

Firstly, this study enriches the related research on the antecedent variables of creative behavior. Compared with a large number of studies on the impact of variables mainly focused on the individual level and different leadership styles on employees’ creative behavior (Chiang et al., 2015), scholars pay less attention to the important role of strategic human resource management at the organizational level. However, the human resource management system implemented by enterprises plays a vital role in shaping individual behavior (Jiang K. et al., 2012; Alqudah et al., 2022). From the existing research results on human resource management practice and employees’ creative behavior, more attention is paid to the impact of high-performance work systems and high-involvement work systems (Liao et al., 2021), but there is relatively little research on the mechanism of high-commitment work systems on employees’ creative behavior, which is different from the above two in management philosophy and management purpose (Xiao and Björkman, 2006). In addition, since they paid more attention to how the high-commitment work systems perceived by employees affects employees’ attitudes and behaviors, scholars in the past studied high-commitment work systems as a variable at the individual level and failed to explore objectively its relationship with employees’ creative behavior as an influencing factor at the organizational level (Ahmed et al., 2018). Therefore, considering the nesting characteristics of environmental factors, this paper selects the high-commitment work systems at the organizational level as the antecedent variable affecting employees’ creative behavior, constructs a multi-level structural equation model, and finds that the high-commitment work systems can significantly predict employees’ creative behavior. This conclusion will help the academic and practical circles further understand the impact of strategic human resource management on employees’ creative behavior.

Secondly, the research conclusion expands the research on the mechanism of high-commitment work systems on employees’ creative behavior. Based on the conservation of resources theory, this study discusses and tests the mediating role of employees’ wellbeing in the impact of high-commitment work systems on employees’ creative behavior. Previously, the impact of high-commitment work systems on employees’ creative behavior was mainly studied from the perspectives of social exchange theory and creativity component theory (Chang et al., 2014; Li et al., 2017). This study proposes that employees may have a sense of wellbeing because of the resources provided by the organization, and positive emotional experience will increase the stock of individual resources, help employees produce creative behavior and realize the spiral gain of resources. Therefore, this study enriches the theoretical perspective of the impact of high-commitment work systems on employees’ creative behavior, better explains the internal mechanism of employees’ creative behavior, and expands the relevant knowledge of conservation of resources theory and its application in enterprise management practice.

Finally, the moderating effect of CEO inclusive leadership on the process of high-commitment work systems affecting employees’ creative behavior through employees’ wellbeing is discussed. From the existing empirical research on high-commitment work systems and employees’ creative behavior, scholars have tested the moderating role of creativity atmosphere, work-life conflict and other factors between them while this study discusses the boundary conditions affecting the relationship between high-commitment work systems and employees’ creative behavior combined with the substitutes for leadership theory (Schimansky, 2014; Chen et al., 2018). On the one hand, it considers the possible effects of CEO’s leadership style on employees at the organizational context level, which is conducive to further understanding the boundary conditions of creative behavior. On the other hand, this study confirmed that the CEO with inclusive leadership style is the environmental condition influencing the effectiveness of high-commitment work systems, which means CEO inclusive leadership will weaken the positive predictive effect of high-commitment work systems on employees’ wellbeing.

### Practical implication

The findings of this study also have important implications for management practice.

In order to effectively motivate employees to creative, the organization should pay full attention to the role of high-commitment work systems at the strategic level, effectively implement the high-commitment work systems, and timely optimize the human resource management system, such as selecting employees with high creativity ability through talent evaluation in the recruitment process; carry out rich and diverse training activities; pay attention to the needs of employees and encourage information sharing and cooperation among employees; pay attention to team building, effectively expand the resource stock of employees, and ensure that employees have sufficient resources to carry out creativity activities.

Secondly, organizations should emphasize employees’ wellbeing, because employees’ wellbeing experience directly reflects employees’ current psychological state, and it is one of the key indicators of high-commitment work systems affecting employees’ creative behavior, which is closely related to the sustainable development of enterprises (Page and Vella-Brodrick, 2009). In the workplace, the acquisition of wellbeing is often closely related to the organization’s human resources policies and practices (Xia et al., 2019). Therefore, for the organization, when optimizing the human resources management system, some policies and measures related to employees’ wellbeing can be added, such as encouraging employees to create and getting equipped with corresponding incentive schemes; timely keep eyes on the psychological state of employees, and provide psychological counseling and other services when negative emotions and other adverse states are found; carry out family day, group counseling, corporate yoga and other activities to improve the happy experience of employees.

Finally, human resource management practice is also affected by CEO leadership styles and other contextual variables in the process of influencing employees’ creative behavior. Based on the research conclusion of substitute effect, if the enterprise’s high commitment human resource management practice cannot achieve the expected effect due to some factors, such as it takes a long time to establish and implement, it becomes extremely important to maximize the positive effect of inclusive leadership style on employees’ attitude and behavior.

## Research limitations and prospects

This study still has some limitations, which we hope to further explore in future researches.

Firstly, in terms of data collection, although this study adopts the form of questionnaire completed by CEO, HR manager and employees, the data collected at the same time point may affect the verification of research assumptions. Therefore, in future research, cross-lagged panel data can be collected in the form of cross time-point to further verify the causal relationship between variables and improve the reliability of research conclusions.

Secondly, although this study discusses the mechanism of high-commitment work systems affecting employees’ creative behavior through employees’ wellbeing, it is worth noting that the meaning of the wellbeing variable used in this paper is relatively broad, drawing on the different types of wellbeing divided by scholars, such as subjective wellbeing and realized wellbeing (Waterman, 1993). Future research can further explore the mediating mechanisms of different types of wellbeing and other possible mediating mechanisms (such as knowledge management) (Lin and Sanders, 2017).

Finally, this study brings CEO inclusive leadership into the research model to explore its boundary role in the impact of high-commitment work systems on employees’ creative behavior, but it still needs to consider other possible moderative variables, such as the impact of individual characteristics, e.g., psychological capital, organizational climate and other contextual factors on the process (Miao and Cao, 2019). In addition, this study focuses on the substitution between CEO’s inclusive leadership and high commitment work system. However, the possible effectiveness of different leaders is different. Take service-oriented leadership as the example. Service-oriented leadership refers to consistently providing high-quality personal services for everyone (including themselves, others, systems, etc.) to meet the needs of all (Shek et al., 2015). The difference between service-oriented leadership and inclusive leadership lie in that service-oriented leadership pays more attention to “serving others,” which is embodied in “service orientation” and “altruism” at the same time when paying attention to the growth of employees. The research of Jiang et al. (2015) shows that service-oriented leadership and service-oriented high-performance work system will reduce each other’s positive impact on collective customer knowledge and service atmosphere. Therefore, in the follow-up research, empirical research can be taken to investigate the relationship between different leadership styles (such as service leadership), especially senior leadership, and enterprise human resource management practice.

## Data availability statement

The raw data supporting the conclusions of this article will be made available by the authors, without undue reservation.

## Author contributions

MZ and LZ: conceptualization. MZ, ZC, and XL: writing—review and editing. MZ: funding acquisition. MZ, LZ, and ZZ: data curation. ZC and XZ: supervision. All authors have read and agreed to the published version of the manuscript.
